# Comparison of Biomarkers of Exposure in a Controlled Study of Smokers Switched from Conventional Cigarettes to Heated Tobacco Products

**DOI:** 10.3390/toxics11100816

**Published:** 2023-09-28

**Authors:** Xiaonan Li, Xuan Wang, Peicai Cui, Guangchao Liu, Hui Zhang, Yihan Gao, Zhenpeng Kai

**Affiliations:** 1 Shanghai New Tobacco Product Research Institute Co., Ltd., Shanghai 201315, China; biolxn@163.com (X.L.); wangxuan@sh.tobacco.com.cn (X.W.); cuipeicai@sh.tobacco.com.cn (P.C.); lgc@sh.tobacco.com.cn (G.L.); zhangh1@sh.tobacco.com.cn (H.Z.); 2 School of Chemical and Environmental Engineering, Shanghai Institute of Technology, Shanghai 201418, China

**Keywords:** heated tobacco product, cigarette, biomarker, urine, toxicant exposure

## Abstract

The heated tobacco product (HTP) heats rather than burns tobacco to release an aerosol with significantly fewer toxicants than conventional cigarette smoke and has received global attention in recent years. To investigate whether changes in biomarkers of exposure could be detected after switching from conventional cigarettes (CCs) to HTPs, 224 subjects from four cities in China participated in this study. Nine biomarkers containing tobacco-specific nitrosamines (TSNAs), volatile organic compounds (VOCs), and the biomarkers for acrolein and crotonaldehyde were determined by UPLC-MS/MS. The levels of the sum of nine biomarkers in CCs were 5.4 and 5.2 times higher than in an Original-HTP and Menthol-HTP, respectively. Among the nine biomarkers, 3HPMA and 3HMPMA accounted for the highest proportions. Switching from CCs to HTPs is good for both men and women because the changes in each biomarker in urine samples were the same in men and women. Among all the subjects, subjects aged 20–39 years had the greatest reduction in biomarker residues in urine. The findings of the present study provided useful information for the health risk research of HTPs in China.

## 1. Introduction

Cigarette smoking has been identified as one of the leading preventable causes of human morbidity and mortality, which are related to the inhalation of a number of toxic chemicals in cigarette smoke [1,2]. More than 6500 chemical components have been found in the smoke generated by tobacco combustion and pyrolysis, of which approximately 150 are established toxicants [3]. The World Health Organization (WHO) Study Group on Tobacco Product Regulation has proposed mandatory lowering of the emission levels from cigarettes of nine specific toxicants: CO, formaldehyde, acetaldehyde, acrolein, 1,3-butadiene, benzene, benzo[a]pyrene, Nnitrosonornicotine (NNN), and 4-(methylnitrosamino)-1-(3-pyridyl)-1-butanone (NNK) [4]. Recently, advances have focused on heating tobacco products (HTPs), which heats rather than burns tobacco. This product releases an aerosol with approximately 90% reduction in toxicants than conventional cigarette smoke [5]. Vukas et al. reported that nicotine delivery by HTPs was significantly lower than that by conventional cigarettes, suggesting a lower addictive potential [6].

The tobacco-specific nitrosamines (TSNAs) are mainly formed and accumulated during the post-harvest processing of tobacco and during flue gas inhalation by nitrosation of nicotine and other tobacco alkaloids. TSNAs are an important part of the harmful and potentially harmful constituents (HPHCs) [7,8]. Therefore, analyzing the concentration of TSNAs in human urine is of great significance for assessing the potential harm of different tobacco products to humans [9]. Volatile organic compounds (VOCs) are an important class of harmful components in cigarette smoke. These substances have a great impact on human health, and many of them have strong carcinogenic effects. VOCs in smoke mainly include acrolein, acrylonitrile, 1,3-butadiene, crotonaldehyde, propylene oxide, styrene, benzene, and toluene. S-phenylmercapturic acid (SPMA) is highly specific as a metabolic marker of benzene. Determination of SPMA, a metabolite of cigarettes in urine, found a positive correlation between smoking and SPMA levels in urine. The concentration of urine SPMA in non-smoking, non-occupational contact people is generally less than 1/5 of smokers, and the concentration of urine SPMA in heavy smokers is 10 times higher than that of ordinary smokers [10]. Monohydroxybutenyl mercapturic acid (MHBMA) and dihydroxybutyl mercapturic acid (DHBMA) are the main metabolites of 1,3-butadiene in the human body and are used as characteristic biomarkers [11]. The International Agency for Research on Cancer (IARC) classifies acrolein as a Class III carcinogen, and Health Canada’s list of harmful components in cigarette smoke and the Hoffmann list also include it [12,13,14]. Acrolein is a highly electrophilic α,β-unsaturated aldehyde, which is found in all types of smoke (including cigarette smoke). At low doses, acrolein inhibits cell proliferation without causing cell death and may enhance apoptosis from secondary toxins, while at higher doses, oncosis ensues [10]. Crotonaldehyde is a α, β-unsaturated carbonyl compound, which can invade the body through the mouth, nose, and skin, causing serious harm to health. The U.S. Environmental Protection Agency (EPA) lists crotonaldehyde as a probable human carcinogen (Group C) based on limited animal data and supporting genotoxicity data [15]. 3-hydroxypropylmercapturic acid (3HPMA) and 3-hydroxy-1-methylpropylmercapturic acid (3HMPMA) are considered specific biomarkers for acrolein and crotonaldehyde, respectively [16,17].

Market research found that a large number of Chinese are interested in HTPs [18]. Despite more and more people being aware of HTPs and having the intention of using them, there have been few studies assessing changes in tobacco biomarkers within Chinese subjects who switched from conventional cigarettes (CCs) to HTPs. The adoption of HTPs in Spain has been like other products considered "healthy", such as additive-free and ultra-slim cigarette brands. The laws should restrict any marketing of tobacco products that promotes positive connotations between tobacco use and being healthy. If left unchecked, it has the potential to lead to the proliferation of smokers, especially younger smokers [19]. Our work was initiated to investigate and document the changes in the levels of nine biomarkers exposure in a study of 224 smokers who switched from CCs to HTPs in four cities in China.

## 2. Materials and Methods

The study was designed and conducted in accordance with the ethical principles of the Declaration of Helsinki and referred to the protocol published by Shepperd et al. [20]. In order to make the research results more representative, this study was conducted in four cities (Shanghai, Suzhou, Hefei, and Guiyang) in China between July and October 2022. This protocol has passed the review of the Ethics Committee before the implementation of the project. The forced-switch clinical trials were conducted by Tongren Traditional Chinese Medicine Hospital, Guizhou, China. All subjects provided written informed consent.

### 2.1. Products

A commercially available conventional cigarette delivered 11 mg tar and 1.0 mg nicotine was used in this study. HTPs with “original-flavor” sticks (original-HTPs) and “mint-flavor” sticks (Menthol-HTPs) were provided by Shanghai New Tobacco Product Research Institute (Nicotine: 1.0 mg/stick; Maximum heating temperature: 350 °C). Interventional studies involving animals or humans and other studies that require ethical approval must list the authority that provided approval and the corresponding ethical approval code.

### 2.2. Participants and Study Groups

A total of 224 regular smokers (who only smoke conventional cigarettes) were enrolled via the study website, word of mouth, phone, and mailings. All subjects were enrolled in the study after inclusion criteria and exclusion criteria had been checked and participants had provided written informed consent. Eligible subjects were healthy adults of any ethnic origin who lived in or around those four cities, as described above. The inclusion and exclusion criteria are referred to in the paper by Shepperd et al. [20]. In this study, smokers had to be aged 20–65 years; no cardiovascular, respiratory, or nervous system disease; no psychiatric and other serious psychiatric disorders; no existing pregnancy or breastfeeding; no medication was taken within the past week; and never smoked HTPs before. Participants had to have smoked 5–20 cigarettes with ISO tar yield 10–12 mg and smoke nicotine level of 1.0 mg daily for at least 2 years. At the same time, they should have smoked the current brand for more than 6 months. Table S2 shows the information of the 224 eligible subjects.

**Figure 1:**
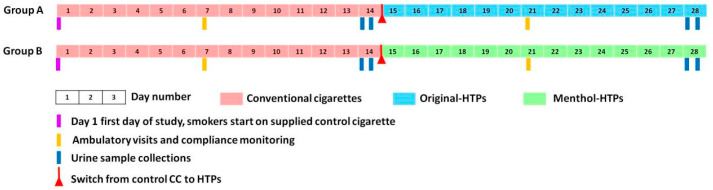
Study design and scheduled events.

This study was conducted independently in the four cities mentioned above. In each city, the subjects were randomly divided into two groups (Group A and Group B). Group A (111 subjects) switched from conventional cigarettes (CCs) to original-HTPs, and Group B (113 subjects) switched from CCs to menthol-HTPs (Figure 1). Each subject smoked only supplied cigarettes during this study.

### 2.3. Study Design

Figure 1 shows the details of the study design and scheduled events. To ensure that enough subjects were recruited for all groups, subject availability was assured, and groups were well matched for age and gender, but full randomization was not possible. On day 1, all subjects received control CCs (supplied by this study) to smoke in their usual manner and daily smoking volume. On day 13, smokers entered the clinic for the first period of urine sample collection. At 8 pm that evening, the subjects were prohibited from smoking any tobacco products. The urine sample (recorded as 0 h) was collected at 8 am on day 14, and then subjects smoked a CC; the second urine sample (24 h urine sample) was collected from subjects for calculation of daily output of urinary biomarkers [21]. Next, the subjects switched to smoking original-HTPs (Group A) or menthol-HTPs (Group B), respectively. Smoking is prohibited after 8 pm on day 27. The urine sample was collected at 8 am on day 28 (recorded as 0 h), followed by an HTP, and the second urine sample was collected throughout the day (recorded as 24 h urine sample), and the clinical trial ended. On days 1, 7, and 21, smokers visited the clinic to collect supplies of cigarettes sufficient for the next ambulatory period. In this study, subjects were asked to smoke a consistent smoking volume each day, whether they smoked CCs or HTPs. During this clinical trial, the subjects were required to record the number of cigarettes smoked and return all the filters. This ensured complete collection of filters and accurate data on cigarette consumption.

### 2.4. Sample Collection and Preparation

All the urine samples from smokers were stored at −40 °C in tubes until analysis for each of the biomarkers. Urine samples were thawed overnight at 4 °C and thoroughly mixed. For the analysis of SPMA, 3-HPMA, 3-HMPMA, DHBMA (*R,S*-1,2-dihydroxy-4-(*N*-acetylcysteinyl)-butane) and MHBMA (a mixture of *R,S*-1-hydroxy-2-(*N*-acetylcysteinyl)-3-butene and *R,S*-2-hydroxy-1-(*N*-acetylcysteinyl)-3-butene), 100 μL of the urine was transferred to a 2 mL centrifuge tube containing 100 μL methanol and 2 ng 3-HPMA-d3 as an internal standard, ultrasonicated at 4 °C for 30 min. After centrifugation at 14,500× *g* for 15 min, the supernatants were filtered through a 0.22-μm syringe filter, and then a volume of 100 μL of the filtrates was introduced into the LC-MS/MS system [22]. Detection of NNN, NAB, NAT and NNAL were according to the published methods. The urine sample was hydrolyzed by β-glucuronidase treatment prior to SPE and LC-MS/MS analysis [23].

### 2.5. Determination of Biomarkers

The methods utilized for measurement of the nine biomarkers have been reported elsewhere [22,23,24,25,26]. The UPLC-MS/MS system consisted of Waters ACQUITY UPLC I-Class (Waters Corp., Milford, MA, USA), coupled with AB SCIEX TRIPLE QUADTM 5500 mass spectrometer from AB Sciex (Framingham, MA, USA). Analyte-specific MS/MS conditions and LC retention times for LC-amenable analytes are shown in Table S3, and the MS source conditions are shown in Table S4. A limit of quantification (LOQ) of 8 ng/mL was obtained for all the 9 biomarkers present in urine, and the correlation coefficients (*r*^2^) were >0.995 within a linearity range of 2–1000 ng/mL.

### 2.6. Data Analysis and Statistics

The residue data were compiled in Microsoft Office Excel 2010. Statistical analyses were performed with GraphPad Prism version 5.0. A value of 0.05 was used as the threshold for significance. Comparisons of biomarker concentrations in the urine samples of the subjects switched from CCs to HTPs were analyzed with a pooled *t*-test.

## 3. Results and Discussion

### 3.1. Comparison of Biomarkers in Urine between CCs and HTPs

**Figure 2:**
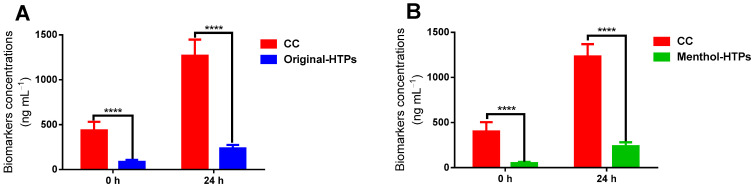
The sum concentrations of 9 biomarkers in the urine samples of the subjects switched from CCs to Original-HTP (**A**) and Menthol-HTP (**B**), respectively. Values represent means ± SD, **** *p* < 0.0001.

The nine biomarkers were detected in all urine samples. Figure 2 shows the sum of nine biomarkers (∑_9_ biomarkers) in each sample of the subjects and compares the differences in the content of harmful substances in the urine samples after smoking CCs and HTPs. In group A, after 12 h of prohibition, the number of biomarkers in urine samples of subjects who smoked CCs was 436.00 ± 95.26 ng/mL, while the total content of biomarkers in samples that smoked Original-HTPs was 85.08 ± 23.63 ng/mL. In group B, the sum of nine biomarkers (∑_9_ biomarkers) in the urine samples recorded as 0 h of the subjects who smoked CCs and Menthol-HTP was 401.73 ± 102.36 ng/mL and 49.70 ± 13.02 ng/mL, respectively. Our results showed that after smoking an HTP for two weeks, the biomarker residues in the urine sample were significantly lower than in the participants who smoked a conventional cigarette after 12 h of abstinence.

After collecting morning urine (recorded as 0 h), subjects smoked a CC or an HTP, and a second urine sample (24 h urine sample) was collected. Figure 2 showed that the levels of the sum of nine biomarkers in CCs were higher than HTPs. It is 5.4 and 5.2 times higher in groups A and B. Our results agree with the study by Shepperd et al. [27] that biomarkers of exposure significantly declined in reduced-toxicant-prototype cigarette (RTP) smokers. The results of Gee et al. also found mouth level exposure to nicotine-free dry particulate matter (NFDPM) and nicotine levels were significantly lower when using HTPs than CCs [28].

**Figure 3:**
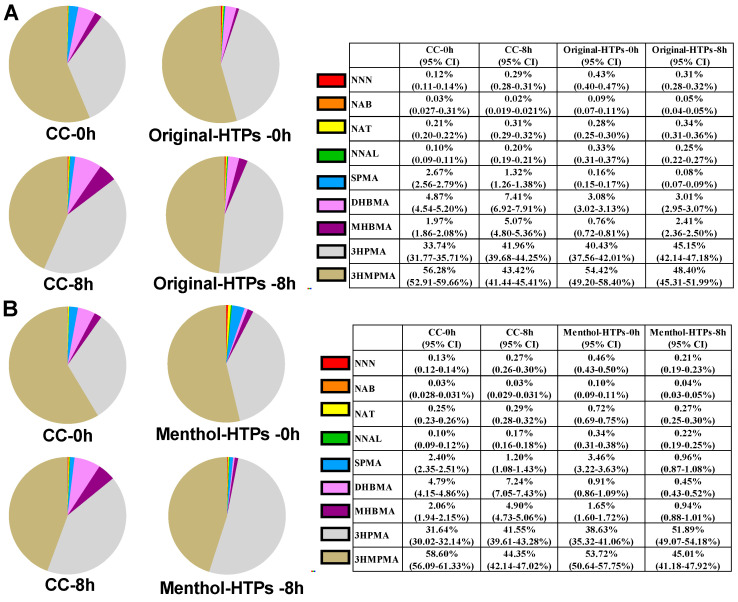
The proportion of each biomarker in the urine samples of the subjects switched from CCs to Original-HTP (**A**) and Menthol-HTP (**B**), respectively. A total of 95% CI: 95% Confidence Intervals.

Among the nine biomarkers, 3HPMA and 3HMPMA accounted for the highest proportions, accounting for 31.64–51.89% and 43.42–58.60%, respectively (Figure 3). The account of VOCs was approximately 2.5–13.8%, while TSNAs have the lowest content, between 0.5 to 1.6%. When subjects switched from CCs to Original-HTP, the proportion of VOCs decreased significantly, while the proportion of TSNAs, 3HPMA, and 3HMPMA increased slightly. In group B (switched from CCs to Menthol-HTP), only the proportion of VOCs decreased significantly, while the proportion of 3HPMA increased slightly, and the proportions of other biomarkers were similar. 

**Figure 4:**
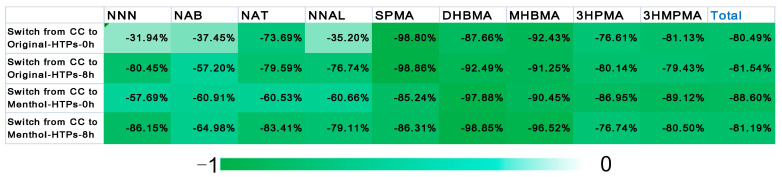
Heat map: show the changes in levels of biomarkers of smokers switched from CCs to HTPs.

The individual concentrations of biomarkers in the urine samples are shown in Table 1. Schaller et al. showed that TSNAs, VOCs, and carbonyl compounds were reduced by at least 90% compared with the mainstream smoke aerosol of CCs [29]. Similarly, our studies have demonstrated that the biomarkers of those above HPHCs in the urine samples were significantly lower than those of CCs. Figure 4 shows the changes in levels of biomarkers (calculated as following Equation (1)) of smokers who switched from CCs to HTPs through the heat maps. In the heat map, a green patch indicated that the biomarker was less in the urine samples of subjects who smoke HTPs than in subjects who smoke CCs. The darker the color, the greater the change in content. After switching to HTPs, the content of all biomarkers decreased significantly, especially the three VOCs. When the subjects switched from CCs to Original-HTP, the contents of SPMA, DHBMA, and MHBMA were 1–10% before switching. Figure 4 showed that the reduction in biomarkers (especially the four TSNAs) content in urine samples after 12 h of prohibition was lower than in urine samples after smoking. The reduction in biomarker residues in urine samples of subjects switched to Menthol-HTP was more significant than that of Original-HTP (*p* < 0.05) (Figure 4). Our results agree with the study by Zhang et al. that when smokers switched from higher to lower TSNA yields of cigarettes, their plasma HPHC levels significantly decreased [30].

Changes = (C_HTP_ − C_CC_)/C_CC_ (1)

where C_HTP_ is the average concentration of a given biomarker in the collected samples of smoked HTPs, and C_CC_ is the average concentration of smoked CCs.

### 3.2. Comparison of Biomarkers between Male and Female

**Figure 5:**
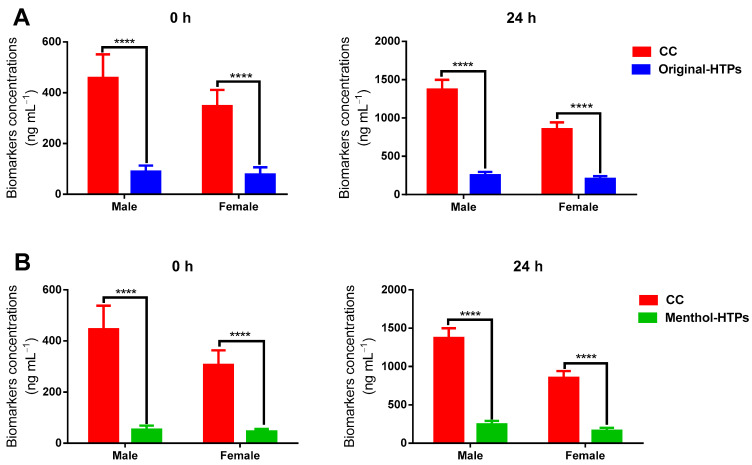
The sum concentrations of 9 biomarkers in male and female switched from CCs to Original-HTP (**A**) and Menthol-HTP (**B**), respectively. Values represent means ± SD, **** *p* < 0.0001.

**Figure 6:**
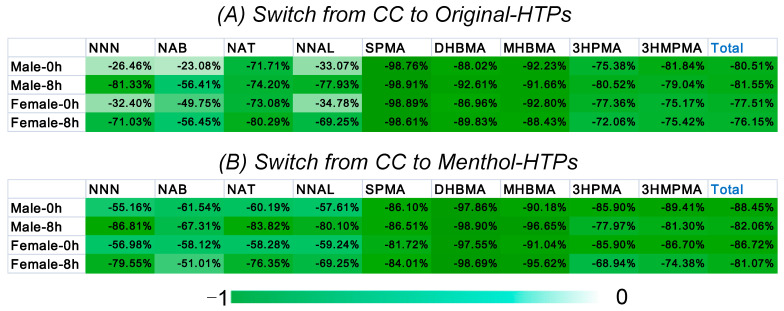
Heat map: show the changes in the content of each biomarker in the male and female samples smokers switched from CCs to Original-HTP (**A**) and Menthol-HTP (**B**), respectively.

Of all the subjects, 179 were male, and 45 were female, a ratio of four to one, which is similar to the ratio of men to women in Chinese urban smokers [18]. Considering that new tobacco products are more attractive to working women, the proportion of women in this study was higher. There were 89 males and 22 females in Group A, while Group B consisted of 90 males and 23 females. Figure 5 shows the sum of nine biomarkers (∑_9_ biomarkers) in males and females and compares the differences in biomarker residues in the urine of males and females when the subjects switched from CCs to HTPs. The biomarkers content in both male and female urine were significantly reduced (*p* < 0.0001). Figure 6 shows the changes (calculated as following Equation (1)) in the content of each biomarker in the male and female samples of smokers who switched from CCs to HTPs. Switching from CCs to HTPs, the levels of biomarkers in urine samples from both men and women were significantly reduced. It suggested that switching HTPs is good for both men and women. 

### 3.3. Comparison of Biomarkers in Subjects of Different Age Groups

**Figure 7:**
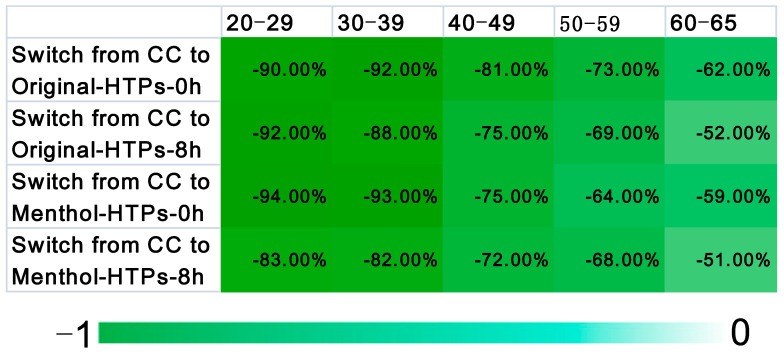
Heat map: show the changes in the sum concentrations of 9 biomarkers in the urine samples of all ages subjects switched from CCs to HTPs.

To explore the effects of switching from CCs to HTPs on people of different ages, we compared the residues of biomarkers in urine samples from subjects of different ages. The subjects of all ages switched from CCs to HTPs with reduced residues of ∑_9_ biomarkers in their urine samples. The reduction in urine biomarker content (calculated as following Equation (1)) in different age groups was presented in Figure 7 through a heat map. The total amount of biomarkers in the urine samples of subjects aged 20–39 years who switched to HTPs was only 10–15% of the previous amount of smoking CCs; the total amount of biomarkers in subjects aged 40–59 years was 20–35% of smoking CCs, and in the urine sample of subjects over 60 years old the total amount was 50–60% before. Our results showed that subjects aged 20–39 years had the greatest reduction in biomarker residues in urine, while those over 60 years had the least reduction in residue amount. There was a significant difference in the reduction in biomarker residues between younger and older subjects (*p* < 0.0001). This might be due to the faster metabolism of young people. We also found that young subjects who switched from CCs to Original-HTP had a greater reduction in urine biomarker levels than those who switched from CCs to Menthol-HTP. A questionnaire survey on the cognitive behavior of HTPs was also conducted. Through this observation, we found that nearly 75 percent of those young subjects generally prefer the Menthol-HTP, so they inhale more smoke from Menthol-HTP. In Asia, interest in HTPs, particularly among young adults, has rapidly increased. Public health research and education on HTPs are needed, especially for the high-risk group [18,31].

All subjects (who only smoked conventional cigarettes) were enrolled in this study after the inclusion criteria and exclusion criteria. Eligible subjects were healthy adults of any ethnic origin who lived in or around those four cities, as described above. However, the reality is far more complicated than that. The need to eradicate tobacco-related health problems and the increasingly complex environments of tobacco research requires sophisticated analytical methods to handle large amounts of data and perform highly specialized tasks. Artificial intelligence and machine learning will help us solve this complex problem [32,33].

## 4. Conclusions

In the present study, the results showed that after smoking HTPs for two weeks, the biomarker residues in the urine sample were significantly lower than in the subjects who smoked a conventional cigarette from the four cities in China. The levels of the sum of nine biomarkers in CCs were 5.4 and 5.2 times higher than in Original-HTP and Menthol-HTP, respectively. Among the nine biomarkers, 3HPMA and 3HMPMA accounted for the highest proportions. After switching to HTPs, the content of all biomarkers decreased significantly, especially the three VOCs. The reduction in biomarker residues in urine samples of subjects switched to Menthol-HTP was more significant than that of Original-HTP. Switching from CCs to HTPs, the changes in each biomarker in urine samples were the same in men and women. It suggested that switching HTPs is good for both men and women. In the comparison of biomarkers in subjects of different age groups, subjects aged 20–39 years had the greatest reduction in biomarker residues in urine, while those over 60 years had the least reduction in residues amount. The findings of the present study provided useful information for the health risk research of HTPs in China.

## Figures and Tables

**Table 1:** Residue concentration of biomarkers detected in urine samples of subjects (ng/mL).

Biomarker	Switched from CCs to Original-HTP (Mean ± SD)				Switched from CCs to Menthol-HTP (Mean ± SD)			
	CCs (0 h)	Original-HTP (0 h)	CCs (24 h)	Original-HTP (24 h)	CCs (0 h)	Menthol-HTP (0 h)	CCs (24 h)	Menthol-HTP (24 h)
NNN	0.54 ± 0.26	0.37 ± 0.13	3.68 ± 1.04	0.72 ± 0.12	0.52 ± 0.31	0.23 ± 0.17	3.28 ± 0.97	0.51 ± 0.16
NAB	0.13 ± 0.05	0.08 ± 0.03	0.26 ± 0.08	0.11 ± 0.03	0.12 ± 0.05	0.05 ± 0.02	0.36 ± 0.06	0.09 ± 0.04
NAT	0.91 ± 0.23	0.24 ± 0.09	3.92 ± 1.02	0.80 ± 0.52	1.01 ± 0.22	0.36 ± 0.20	3.52 ± 0.89	0.65 ± 0.13
NNAL	0.43 ± 0.16	0.28 ± 0.13	2.54 ± 0.73	0.59 ± 0.13	0.40 ± 0.09	0.17 ± 0.06	2.14 ± 0.67	0.53 ± 0.22
SPMA	11.65 ± 2.41	0.14 ± 0.03	16.73 ± 4.59	0.19 ± 0.06	9.65 ± 3.06	1.72 ± 0.15	14.73 ± 3.85	2.29 ± 0.68
DHBMA	21.24 ± 7.31	2.62 ± 1.06	93.90 ± 30.05	7.05 ± 1.72	19.24 ± 6.84	0.45 ± 0.12	89.20 ± 28.95	1.08 ± 0.31
MHBMA	8.59 ± 1.30	0.65 ± 0.24	64.31 ± 18.27	5.63 ± 1.37	8.29 ± 0.98	0.82 ± 0.42	60.31 ± 21.16	2.24 ± 0.45
3HPMA	147.10 ± 58.04	34.40 ± 14.13	531.70 ± 207.28	105.60 ± 33.62	127.10 ± 46.38	19.20 ± 9.17	511.70 ± 196.50	123.70 ± 35.08
3HMPMA	245.40 ± 103.07	46.30 ± 26.92	550.20 ± 193.51	113.20 ± 42.10	235.40 ± 97.64	26.70 ± 13.12	546.20 ± 129.04	107.30 ± 48.02
∑Biomarkers	436.00 ± 95.26	85.08 ± 23.63	1267.24 ± 180.63	233.89 ± 40.17	401.73 ± 102.36	49.70 ± 13.02	1231.44 ± 137.82	238.39 ± 43.16

## Data Availability

The data presented in this study are available in the Supplementary Materials.

## Supplementary Materials

The following supporting information can be downloaded at: https://www.mdpi.com/article/10.3390/toxics11100816/s1, Table S1: Biomarkers in this study; Table S2: The information of the 224 eligible subjects; Table S3: Retention time (RT) and MRM condition of biomarkers for LC-MS/MS analysis. Table S4: The MS source conditions.
