# Supplementary material for: Comparison of Biomarkers of Exposure in a Controlled Study of Smokers Switched from Conventional Cigarettes to Heated Tobacco Products
Source: Toxics. 2023 Sep 28;11(10):816. doi: 10.3390/toxics11100816 (PMC10611248; doi:10.3390/toxics11100816)
Supplement: Supplementary file 1 [file toxics-11-00816-s001.zip › toxics-2567241-supplementary.pdf]

SUPPORTING MATERIALS

Table S1. Biomarkers in this study

Biomarker	Abbreviation
N'-nitrosonornicotine	NNN
N-nitrosoanabasine	NAB
N-nitrosoanatabine	NAT
4-(methylnitrosamino)-1-(3-pyridyl)-1-butanol	NNAL
S-phenylmercapturic acid	SPMA
monohydroxybutenyl mercapturic acid	MHBMA
dihydroxybutyl mercapturic acid	DHBMA
3-hydroxypropylmercapturic acid	3HPMA
3-hydroxy-1-methylpropylmercapturic acid	3HMPMA

Table S2. The information of the 224 eligible subjects

Number of subjects in different cities			
Shanghai	60	Suzhou	58
Hefei	56	Guiyang	50
Number of male and female			
Male	179	Female	45
Number of subjects in different age groups			
20-29	37	30-39	61
40-49	53	50-59	42
60-65	31		

Table S3. Retention time (RT) and MRM condition of biomarkers for LC-MS/MS analysis.

Analyte	tR(min)	Q1	Q3	DP	CE	CXP
NNN_1*	1.83	178.1	148.1	30	15	9
NNN_2	1.83	178.1	119.1	30	15	9
NAB*	2.54	192.1	162.1	26	18	11
NAT*	2.52	190.1	160.1	28	16	10
NNAL_1*	1.76	210	105.7	34	19	3
NNAL_2	1.76	210	131	34	19	3
NNAL_3	1.76	210	148.7	34	9	3
NNN-d4_1*	1.83	182.1	152.1	30	16	9
NNN-d4_2	1.83	182.1	124.1	30	16	9

SPMA*	2.79	237.9	108.9	-43	-15	-10
MHBMA_1*	2.15	232	102.9	-40	-15	-10
MHBMA_2	2.15	232	199	-45	-20	-10
DHBMA_1*	1.21	249.9	120.9	-35	-16	-10
DHBMA_2	1.21	249.9	127.9	-35	-16	-10
3-HPMA_1*	1.49	219.9	90.9	-45	-16	-10
3-HPMA_2	1.49	219.9	84	-45	-17	-10
3-HMPMA_1*	2.34	234	105	-30	-21	-10
3-HMPMA_2	2.34	234	102.9	-20	-39	-10
3-HPMA-d3*	1.49	222.8	90.9	-45	-16	-10

* The ion transitions is for quantitation.

Table S4. The MS source conditions

Ion source	ESI
Curtain Gas	20 arb
Collision Gas	9 arb
IonSpray voltage	4200V
Temperature	450 ℃
IonSource Gas1	35 arb
IonSource Gas2	35 arb
